# PyWGCNA: a Python package for weighted gene co-expression network analysis

**DOI:** 10.1093/bioinformatics/btad415

**Published:** 2023-07-03

**Authors:** Narges Rezaie, Farilie Reese, Ali Mortazavi

**Affiliations:** Department of Developmental and Cell Biology, UC Irvine, Irvine, CA 92697, United States; Center for Complex Biological Systems, UC Irvine, Irvine, CA 92697, United States; Department of Developmental and Cell Biology, UC Irvine, Irvine, CA 92697, United States; Center for Complex Biological Systems, UC Irvine, Irvine, CA 92697, United States; Department of Developmental and Cell Biology, UC Irvine, Irvine, CA 92697, United States; Center for Complex Biological Systems, UC Irvine, Irvine, CA 92697, United States

## Abstract

**Motivation:**

Weighted gene co-expression network analysis (WGCNA) is frequently used to identify modules of genes that are co-expressed across many RNA-seq samples. However, the current R implementation is slow, is not designed to compare modules between multiple WGCNA networks, and its results can be hard to interpret as well as to visualize. We introduce the PyWGCNA Python package, which is designed to identify co-expression modules from large RNA-seq datasets. PyWGCNA has a faster implementation than the R version of WGCNA and several additional downstream analysis modules for functional enrichment analysis using GO, KEGG, and REACTOME, inter-module analysis of protein–protein interactions, as well as comparison of multiple co-expression modules to each other and/or external lists of genes such as marker genes from single cell.

**Results:**

We apply PyWGCNA to two distinct datasets of brain bulk RNA-seq from MODEL-AD to identify modules associated with the genotypes. We compare the resulting modules to each other to find shared co-expression signatures in the form of modules with significant overlap across the datasets.

**Availability and implementation:**

The PyWGCNA library for Python 3 is available on PyPi at pypi.org/project/PyWGCNA and on GitHub at github.com/mortazavilab/PyWGCNA. The data underlying this article are available in GitHub at github.com/mortazavilab/PyWGCNA/tutorials/5xFAD_paper.

## 1 Introduction

Weighted gene co-expression network analysis (WGCNA) is a widely used method for describing the correlation patterns of genes across a large set of samples (Langfelder and Horvath 2008). WGCNA can be used to find modules of highly correlated genes, to summarize modules, to relate modules to one another as well as to external traits, and calculate module membership. Correlation networks facilitate network-based gene screening methods that can be used to identify candidate biomarkers or therapeutic targets. These methods have been successfully applied in many biological contexts, such as cancer, mouse genetics, and analysis of human data. The WGCNA package (Langfelder and Horvath 2008) is implemented in the popular R language. As sequencing datasets grow larger and more complex, it is important to have a scalable implementation of WGCNA.

**Figure 1.**
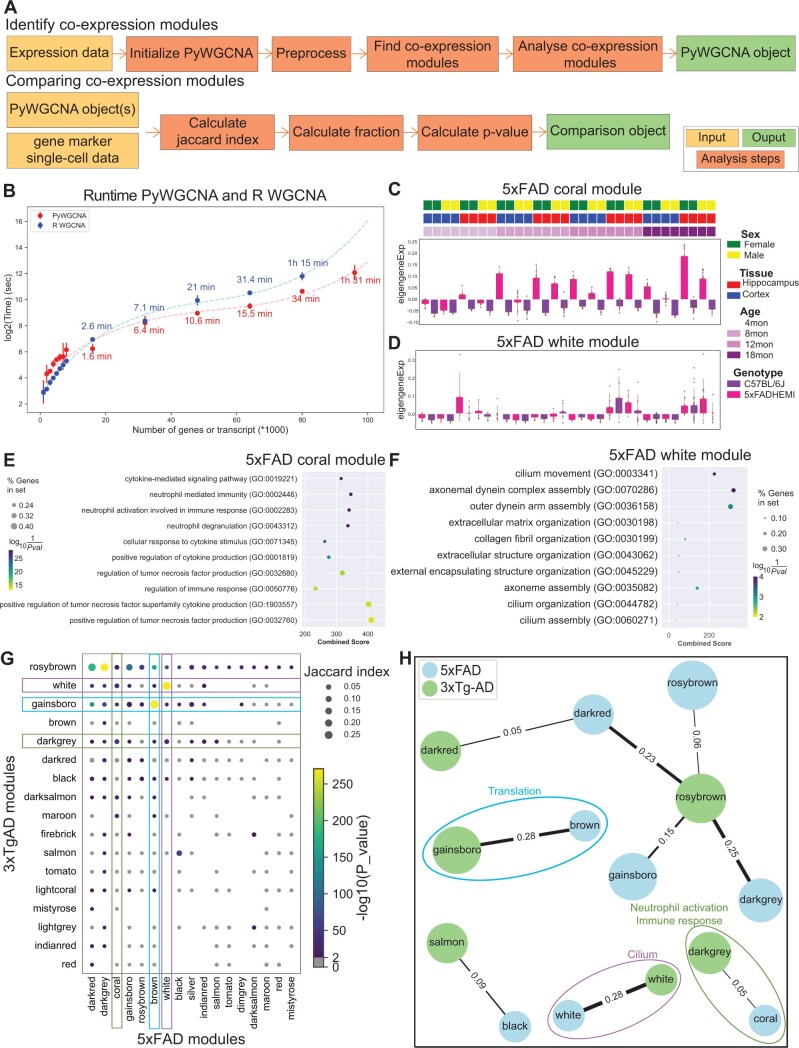
PyWGCNA steps and output example. (A) Overview of PyWGCNA. (B) Average runtime of PyWGCNA and R version of WGCNA versus the number of features used in downsampled datasets in triplicate. (C) Coral and (D) white module eigengene expression profile from 5xFAD mouse model summarized by genotype. Above, the top three rows display the metadata for each dataset including sex, tissue, and age. Below, the bar plot represents module eigengene expression by genotype for each dataset with individual sample module eigengene expression shown as points. GO analysis of the genes in 5xFAD (E) coral and (F) white modules, respectively. (G) Bubble plot of module overlap test results between 5xFAD and 3xTg-AD mouse models of familial AD. The dot size represents the fraction of shared genes between each pair of modules and non-gray color denotes the significance of the overlap between modules. (H) Comparison graph of 5xFAD and 3xTg-AD modules mouse models of familial AD for those with >0.05 Jaccard similarity. The thickness of lines shows the Jaccard index value.

We introduce PyWGCNA, which is designed to perform WGCNA and downstream analytical tasks natively in Python (Fig. 1A). PyWGCNA supports co-expression network analysis of large, high-dimensional gene or transcript expression datasets that are time or memory inefficient in R. PyWGCNA can directly perform functional enrichment analysis including Gene Ontology (GO), KEGG, and REACTOME on co-expression modules to characterize the functional activity of each module. PyWGCNA also supports addition or removal of data to allow for iterative improvement on network construction as new samples become available or need to be taken out. Finally, PyWGCNA can compare co-expression modules from multiple networks with one another to assess module reproducibility or with marker genes from scRNA-seq clusters to assess functional activity or cell-type specificity of each module. We demonstrate PyWGCNA’s utility in identifying co-expression modules associated with genotype in bulk RNA-seq from MODEL-AD (www.model-ad.org) using 5xFAD and 3xTg-AD mouse models of Alzheimer’s disease (AD) and matching WT mice.

## 2 Materials and methods

### 2.1 Identifying co-expression modules

#### 2.1.1 Data preprocessing and initialization of the PyWGCNA object

The PyWGCNA object stores user-specified network parameters such as the network type and major outputs such as the adjacency matrix. PyWGCNA can be initialized from data in csv, tsv or AnnData (Virshup *et al.* 2021) format. We recommend prior preprocessing and normalization of input gene or transcript expression data, including any necessary batch correction. Gene/transcript expression data and metadata about each gene or sample are stored within the object in AnnData format. PyWGCNA can remove overly sparse genes/transcripts or samples and lowly expressed genes/transcripts, as well as outlier samples based on hierarchical clustering and user-defined thresholds.

#### 2.1.2 Finding co-expression modules

PyWGCNA follows an identical approach to the reference WGCNA R package, differing only in default parameter choices. First, PyWGCNA constructs a co-expression matrix by calculating the correlation between each pair of genes/transcripts from the preprocessed expression data. It then constructs a co-expression network based on soft power thresholding the correlation matrix followed by computing the topological overlap matrix to produce the final network. Finally, PyWGCNA identifies co-expressed modules of genes/transcripts by hierarchically clustering the network and performing a dynamic tree cut.

#### 2.1.3 Downstream analysis and visualization of co-expression modules

PyWGCNA provides several options for downstream analysis and visualization of co-expression modules. It can perform module-trait correlation, compute and summarize module eigengene expression across sample metadata categories, detecting hug genes in each module, and perform functional enrichment analysis in each module using databases such as GO, KEGG, and REACTOME (Gillespie *et al.* 2022) via GSEApy (Fang *et al.* 2023) and BioMart (Cunningham *et al.* 2022). PyWGCNA can also recover known and predicted protein–protein interactions within each module using the STRING database (Szklarczyk *et al.* 2021). Each of these analysis options comes with easy-to-use plotting tools to visualize the results. Additional plotting tools include interactive module network visualization with options for selecting genes to display in each module.

### 2.2 Assessing co-expression module overlap between PyWGCNA objects or to single-cell data

PyWGCNA can compare co-expression modules from multiple PyWGCNA objects by computing the Jaccard similarity coefficient and the proportion of common genes for each pair of modules between objects. The statistical significance of the overlap is assessed using Fisher’s exact test. Using the same strategy, PyWGCNA can find the overlap between co-expression modules and different gene lists such as marker genes from single-cell RNA-seq, which can reveal the cell-type specificity of each co-expression module. In both cases, the results from these tests can be easily visualized using PyWGCNA (Fig. 1A).

## 3 Results

In order to compare the performance of PyWGCNA and the R reference of WGCNA, we used expression data for both gene-level (from bulk short-read RNA-seq) and transcript-level (from bulk long-read RNA-seq) expression datasets of 100 samples from the ENCODE portal (www.encodeproject.org). We produced 15 subset datasets with a reduced number of genes or transcripts to calculate how runtime changes as the number of features increases. For each subset, we ran PyWGCNA and R WGCNA three times each on the same number of CPUs (32 cores) and memory allocation (300 GB). We found that while both packages had similar performance up to 16 000 genes, PyWGCNA was twice as fast on larger datasets. Furthermore, we were able to identify modules for 96 000 transcripts with PyWGCNA but were not able to compute a co-expression network in R with the same dataset due to memory constraints (Fig. 1B).

We then applied PyWGCNA to 192 bulk RNA-seq samples of cortex and hippocampus of the 5xFAD mouse model and matching C57BL/6J mice at four ages (4, 8, 12, and 18 months) in both sexes (Forner *et al.* 2021). PyWGCNA recovered 17 gene co-expression modules that are associated with age, genotype, tissue, and sex. The coral module is strongly correlated with age progression in the 5xFAD genotype (*P*-value < 0.05) as illustrated by the module eigengene expression (Fig. 1C), while the white module is significantly correlated with the hippocampus in both genotypes (Fig. 1D). The 5xFAD coral module contains 1335 genes, which are significantly enriched for GO terms related to immune response and neutrophil activation (Fig. 1E). This module includes well-known microglial activation genes such as Cst7, Tyrobp, and Trem2. In contrast, the 5xFAD white module (435 genes) was enriched in GO terms such as cilium movement, organization, and assembly (Fig. 1F).

We also applied PyWGCNA to 38 bulk RNA-seq hippocampus female samples from the 3xTg-AD mouse model and matching WT mice at three ages (4, 12, and 18 months) (Javonillo *et al.* 2022). This analysis yielded 17 modules that are correlated with age or genotype. The dark gray module (380 genes) is strongly correlated with the 3xTg-AD genotype mice and the 18-month time point. GO analysis reveals that this module is also significantly enriched for genes related to neutrophil degranulation and immune response with genes such as Csf1, Tyrobp, and Trem2.

To assess the similarity between modules found in the 5xFAD and 3xTg-AD experiments, we used PyWGCNA to perform module overlap tests. We find several modules with significant overlap that are also enriched for similar functions (Fig. 1G and H). As expected, based on their functional enrichment, the 5xFAD coral module and the 3xTg-AD dark gray module significantly overlap one another, suggesting that the co-expression network within these modules is conserved across the two familial AD mouse models. The 3xTg-AD white module (434 genes) has a strong correlation with 18-month samples and is also enriched in cilium movement. This module significantly overlaps with the 5xFAD white module (Fig. 1H).

## 4 Summary

We have developed a Python package based on the original R implementation of WGCNA. PyWGCNA is capable of handling larger datasets and provides an additional set of well-documented functions. There are several additional downstream functions for the analysis and visualization tools for results such as functional enrichment analysis and identifying protein–protein interactions, as well as multi ways comparison between multiple PyWGCNA networks or with any other gene list. For example, the PyWGCNA Jaccard similarity-based gene list overlap test allows for associating specific cell types to individual modules for further interpretation of the possible functions of these modules. We expect such comparative analyses to grow as the number of datasets grows exponentially. We hope that this package will fill a gap in the Python bioinformatics community.
